# Identification of Viruses and Viroids Infecting Tomato and Pepper Plants in Vietnam by Metatranscriptomics

**DOI:** 10.3390/ijms21207565

**Published:** 2020-10-13

**Authors:** Hoseong Choi, Yeonhwa Jo, Won Kyong Cho, Jisuk Yu, Phu-Tri Tran, Lakha Salaipeth, Hae-Ryun Kwak, Hong-Soo Choi, Kook-Hyung Kim

**Affiliations:** 1 Research Institute of Agriculture and Life Sciences, Seoul National University, Seoul 08826, Korea; bioplanths@snu.ac.kr (H.C.); yeonhwajo@gmail.com (Y.J.); wonkyong@gmail.com (W.K.C.); 2 Plant Genomics and Breeding Institute, Seoul National University, Seoul 08826, Korea; mago03@snu.ac.kr; 3 Department of Agricultural Biotechnology, Seoul National University, Seoul 08826, Korea; tranphutri@gmail.com; 4 School of Bioresources and Technology, King Mongkut’s University of Technology Thonburi, Bangkok 10150, Thailand; lakha.sal@kmutt.ac.th; 5 Crop Protection Division, National Institute of Agricultural Sciences, Rural Development Administration, Wanju 55365, Korea; hrkwakhahn@korea.kr (H.-R.K.); hschoi@korea.kr (H.-S.C.)

**Keywords:** metatranscriptomics, pepper, tomato, Vietnam, virus

## Abstract

Tomato (*Lycopersicum esculentum* L.) and pepper (*Capsicum annuum* L.) plants belonging to the family *Solanaceae* are cultivated worldwide. The rapid development of next-generation sequencing (NGS) technology facilitates the identification of viruses and viroids infecting plants. In this study, we carried out metatranscriptomics using RNA sequencing followed by bioinformatics analyses to identify viruses and viroids infecting tomato and pepper plants in Vietnam. We prepared a total of 16 libraries, including eight tomato and eight pepper libraries derived from different geographical regions in Vietnam. We identified a total of 602 virus-associated contigs, which were assigned to 18 different virus species belonging to nine different viral genera. We identified 13 different viruses and two viroids infecting tomato plants and 12 viruses and two viroids infecting pepper plants with viruses as dominantly observed pathogens. Our results showed that multiple infection of different viral pathogens was common in both plants. Moreover, geographical region and host plant were two major factors to determine viral populations. Taken together, our results provide the comprehensive overview of viral pathogens infecting two important plants in the family *Solanaceae* grown in Vietnam.

## 1. Introduction

Tomato (*Lycopersicum esculentum* L.) and pepper (*Capsicum annuum* L.) plants belonging to the family Solanaceae are cultivated worldwide. Tomato fruit, classified botanically as a berry, is consumed in various ways, such as fresh in salad or as materials for diverse dishes, sauces, and drinks [1,2]. Pepper fruits of Capsicum plants have diverse names according to their regions and types. For instance, piquant pepper varieties are referred as chili peppers, whereas peppers with large- or mid-sized fruits are referred to as bell peppers. Sometimes, colors are important factors for determining names of peppers, such as green pepper and red pepper [3].

To date, diverse viruses infecting tomato and peppers have been reported around the world. Virus infections on tomatoes and peppers have a negative impact on these crops, such as low quality and quantity of fruit production [4,5,6,7,8]. Among the viruses infecting solanaceous vegetables, cucumber mosaic virus (CMV) in the genus *Cucumovirus* might be the most important virus for plants in the family *Solanceae* [9]. Tomato spotted wilt virus (TSWV) in the genus *Tospovirus* is a recently emerging plant virus that causes serious viral diseases in a wide range of important plants, including pepper and tomato [10]. In addition, tomato yellow leaf curl virus (TYLCV) in the genus *Begomovirus* causes severe damage on tomato cultures [11]. Moreover, several viroids, such as citrus exocortis viroid (CEVd), columnea latent viroid (CLVd), pepper chat fruit viroid (PCFVd), potato spindle tuber viroid (PSTVd), tomato chlorotic dwarf viroid (TCDVd) and tomato apical stunt viroid (TASVd) in the family *Pospiviroid*, infect pepper and tomato plants [9].

The rapid development of next-generation sequencing (NGS) technology facilitates the identification of viruses and viroids infecting plants [12,13,14]. NGS-based libraries have been prepared using various methods including small RNAs, double-stranded RNAs, messenger RNA, and ribosomal RNA-depleted total RNA. Hordeum vulgare endornavirus (HvEV), composed of dsRNA as a genome, was identified from barley using MiSeq [15]. In addition, barley yellow striate mosaic virus (BYSMV), a genus of *Cytorhabdovirus*, was identified and assembled into a complete genome using small RNA sequencing analysis [16]. NGS-based approaches reveal not only known viral pathogens, but also novel viral pathogens. In addition, the assembly of the viral genome, viral abundance, and virus mutation can be achieved using NGS-based approaches followed by intensive bioinformatics analyses.

Several viruses infecting tomato and pepper plants in Vietnam have been reported previously [4,17,18,19,20,21,22]. However, most of the reported viruses infecting tomato and pepper in Vietnam have been limited to begomoviruses. Hence, the knowledge of RNA viruses and viroids infecting tomato and pepper plants is very limited. In this study, we carried out metatranscriptomics using RNA-sequencing (RNA-Seq) followed by bioinformatics analyses to identify viruses and viroids infecting tomato and pepper plants in Vietnam. We addressed the diversity of viruses and viroids infecting the two plants in different geographical regions in Vietnam.

## 2. Results

### 2.1. Collection of Leaf Samples and Generation of Libraries for Identification of Viruses Infecting Tomato and Pepper Plants

**Figure 1:**
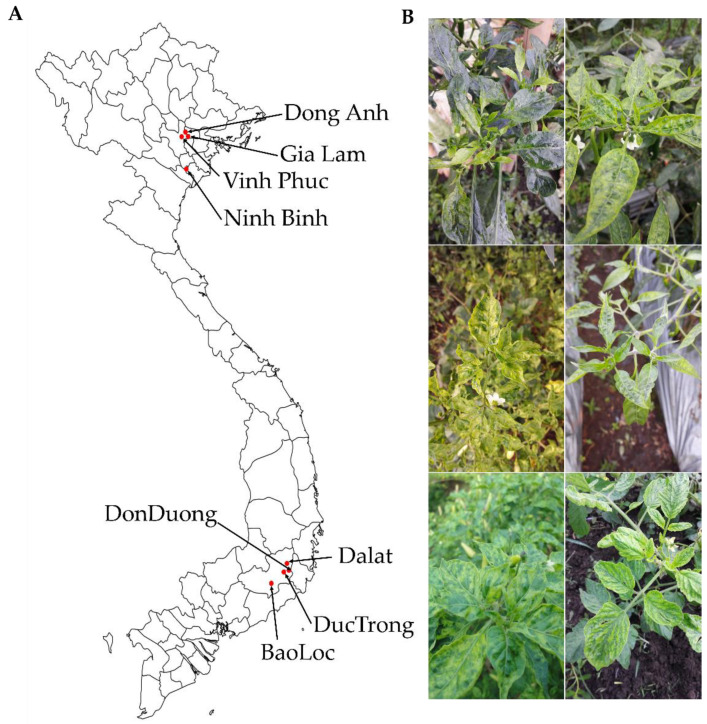
Locations of pepper and tomato sample collection (**A**) and typical symptoms observed in collected samples (**B**).

To identify viruses infecting tomato and pepper plants, we collected leaf samples showing viral disease symptoms, including yellowing, mosaic, mottling, and dwarfing, in different geographical regions in Vietnam (Figure 1). We collected samples from eight and five different regions for tomatoes and peppers, respectively. In the case of pepper samples, three different kinds of pepper such as chili pepper, bell pepper, and red pepper were collected. For the pooling of the samples, we collected more than three leaves from each farm located in each individual region.

Samples collected from the same host plants in the same geographical regions were pooled and subjected to total RNA extraction followed by preparation of libraries for RNA-Seq. We named each library according to geographical regions and host plants. For example, the tomato sample collected from Dong Ahn was named DAT (Dong Ahn Tomato) and the chili pepper sample collected from Gia Lam was named GLCP (Gia Lam Chili Pepper). In total, we prepared 16 libraries, including eight tomato and eight pepper libraries. The prepared libraries were paired-end (2 × 100 bp) sequenced by HiSeq 2000 system.

### 2.2. Identification of Virus-Associated Contigs

**Figure 2:**
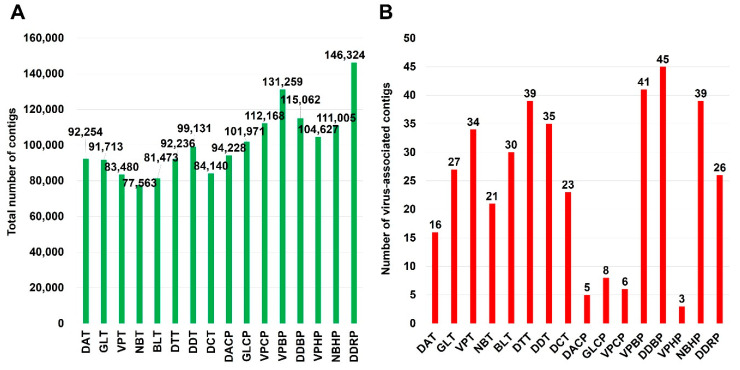
Number of assembled contigs and virus-associated contigs. (**A**) The total number of contigs assembled by Trinity program in each library. (**B**) The number of virus-associated contigs in each library, which was identified by BLASTN search against viral genome database.

The numbers of raw read bases ranged from 8,473,848,288 (DDBP library) to 4,899,434,654 (NBT library). The sequenced raw data of each library were assembled using the Trinity program for de novo assembly. The number of assembled contigs ranged from 77,563 (library NBT) to 146,324 (DDRP library) (Figure 2A).

To identify virus-associated contigs, we performed a BLASTN search using assembled contigs against virus reference genome database obtained from NCBI database. We identified a total of 602 virus-associated contigs. The number of virus-associated contigs for tomato (450 contigs) was higher than that for pepper (152 contigs) (Figure 2B). The number of identified virus-associated contigs was ranged from three (VPHP) to 45 (DDBP).

### 2.3. Classification of Identified Virus-Associated Contigs According to Virus Taxonomy

We identified 18 different virus species belonging to nine different viral genera (Supplementary Table S1). We identified Potato virus Y (PVY) and Chilli veinal mottle virus (ChVMV) belonging to the genus *Potyvirus*. Three viruses in the genus *Tobamovirus*—tomato mosaic virus (ToMV), pepper mild mottle virus (PepMMV), and tobacco mild green mosaic virus (TMGMV)—were identified in both plants. In addition, CMV and pepper vein yellow virus (PepVYV) from the genus *Polerovirus* were identified. Interestingly, we identified three viruses with tripartite RNA fragments such as capsicum chlorosis virus (CaCV), pepper chlorsotic spot virus (PCSV), and tomato necrotic ring virus (TNRV) from the genus *Tospovirus*. We identified four different single-stranded DNA viruses such as ageratum yellow vein China virus (AYVCNV), tomato yellow leaf curl Kanchanaburi virus (TYLCKaV), lindernia anagallis yellow vein virus (LaYVV), and tomato yellow leaf curl Vietnam virus (TYLCVNV) from the genus *Begomovirus*. Moreover, two double-stranded (ds) RNA viruses—pepper cryptic virus 2 (PCV2) in the genus *Deltapartitivirus* and southern tomato virus (STV), from the genus *Amalgavirus*—were identified. Furthermore, two viroids, CLVd and PCFVd from the genus *Pospiviroid* were identified.

### 2.4. Proportion of Identified Viruses and Viroids According to Virus-Associated Contigs

**Figure 3:**
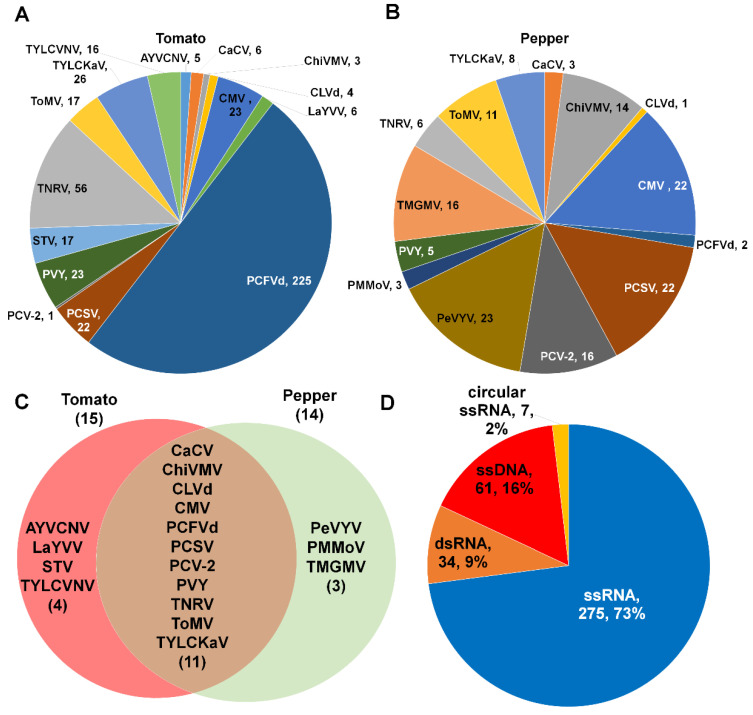
Number of virus-associated contigs for individual identified viral pathogen. The pie charts display the number of virus-associated contigs for identified viruses and viroids infecting tomato (**A**) and pepper (**B**) plants. (**C**) Venn diagram displays a comparison of identified viral pathogens between tomato and pepper plants. Full virus names can be found in Supplementary Table S1. (**D**) Proportion of identified viral pathogens according to viral genome type.

We identified 13 different viruses and two viroids from eight different tomato libraries. Of these, PCFVd (225 contigs) was the most frequently identified viral pathogen based on the number of identified contigs, followed by TNRV (56 contigs), TYLCKaV (26 contigs), PVY (23 contigs), and CMV (23 contigs) (Figure 3A).

In the case of pepper, we identified 12 viruses and two viroids. PeVYV (23 contigs) was the most frequently identified virus, followed by CMV (22 contigs), PCSV (22 contigs), PCV-2 (16 contigs), and TMGMV (16 contigs) (Figure 3B). We compared the identified viruses and viroids between tomato and pepper (Figure 3C). Nine viruses and two viroids (CLVd and PCFVd) were commonly identified in both plants. AYVCNV, LaYVV, STV, and TYLCVNV were identified specifically in tomato while PeVYV, PMMoV, and TMGMV were identified only in pepper. According to viral genome types, the most identified virus-associated contigs were derived from single-stranded (ss) RNA genome (73%), followed by ssDNA genome (16%), dsRNA genome (9%), and circular ssRNA genome (2%) (Figure 3D).

### 2.5. Proportion of Identified Viruses and Viroids According to Virus-Associated Reads

**Figure 4:**
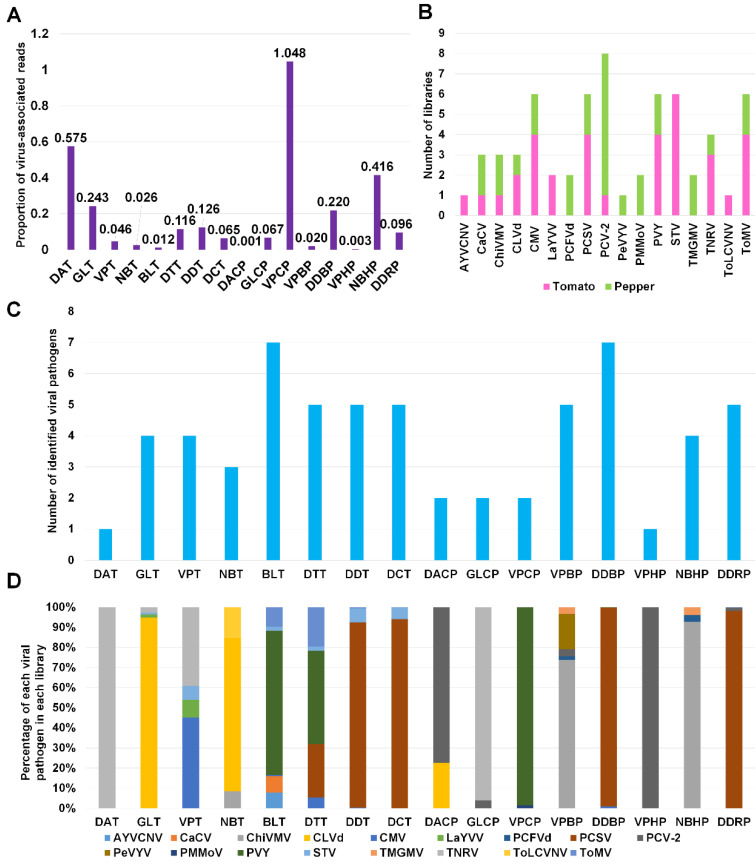
The viral population of identified viral pathogens in each library. (**A**) The proportion of virus-associated reads in each library. (**B**) The frequency of identified viral pathogens in different tomato and pepper libraries. Purple- and green-colored bars indicate the number of libraries for tomato and pepper plants, respectively, in which viral pathogens were infected. (**C**) The number of identified virus and viroid species in each library. (**D**) The viral population of identified viral pathogens in each library based on the number of virus-associated reads.

Next, we calculated the proportion of virus-associated reads in each library (Figure 4A). Except VPCP (1.048%), the proportion of virus-associated reads was less than 1% in all libraries. For eight tomato libraries, the proportion of virus-associated reads ranged from 0.012% (BLT) to 0.575% (DAT), whereas the proportion of virus-associated reads for eight pepper libraries ranged from 0.001% (DACP) to 1.048% (VPCP).

We examined the number of libraries for each virus and viroid (Figure 4B). PCV-2 was identified in eight libraries (one tomato and seven pepper libraries). AYVCNV was identified only in a single tomato library, while PeVYV and ToLCVV were identified only in a single pepper and tomato library, respectively. In contrast to other viruses, STV was identified in six tomato libraries.

We examined the number of identified viral pathogens in each library (Figure 4C). Except two libraries, DAT and VPHP, all libraries contained at least two different viral pathogens. Of these, two libraries, BLT and DDBP, contained at least seven different viral pathogens. Three tomato libraries, DTT, DDT, and DCT, and a single pepper library, DDRP, were infected by five different viral pathogens.

Based on virus-associated reads, we examined the proportion of viral pathogens in each library (Figure 4D). TNRV was the dominant virus in DAT and GLCP. CLVd was the dominant viral pathogen in GLT and NBT. PVY was the dominant virus in BLT, DTT, and VPCP. In DDT, DCT, DDBP, and DDRP, PCSV was the dominant virus. PCV-2 was dominantly present in DACP and VPHP. ChiVMV was dominant in VPBP and NBHP.

### 2.6. Proportion of Identified Viruses and Viroids According to Plant Host and Geographical Region

**Figure 5:**
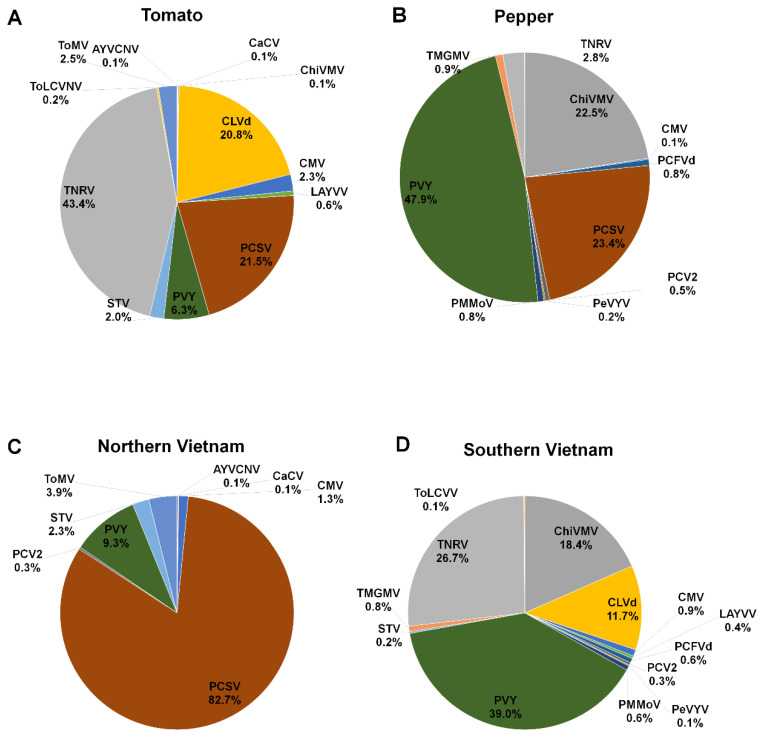
The proportion of identified viral pathogens according to host plants and geographical regions. Pie charts display proportion of identified viral pathogens in tomato (**A**) and pepper (**B**) plants according to virus-associated reads. The proportion of identified viral pathogens in northern (**C**) and southern Vietnam (**D**) based on virus-associated reads.

According to plant hosts, we examined the proportion of identified viral pathogens by combining all virus-associated reads. In tomato plants, TNRV (43.4%) was the most dominant viral pathogen, followed by PCSV (21.5%) and CLVd (20.8%) (Figure 5A). In pepper plants, PVY (47.9%) was the dominant virus, followed by PCSV (23.4%) and ChiVMV (22.5%) (Figure 5B). We next examined virus proportion according to Northern and Southern Vietnam, regardless of plant hosts. In Northern Vietnam, PCSV (82.7%) was the dominant viral pathogen, followed by PVY (9.3%) and ToMV (3.9%) (Figure 5C). In Southern Vietnam, PVY (39%) was the dominant viral pathogen, followed by TNRV (26.7%), ChiVMV (18.4%), and CLVd (11.7%) (Figure 5D).

### 2.7. Phylogenetic Analyses for Identified Viruses and a Viroid

**Figure 6:**
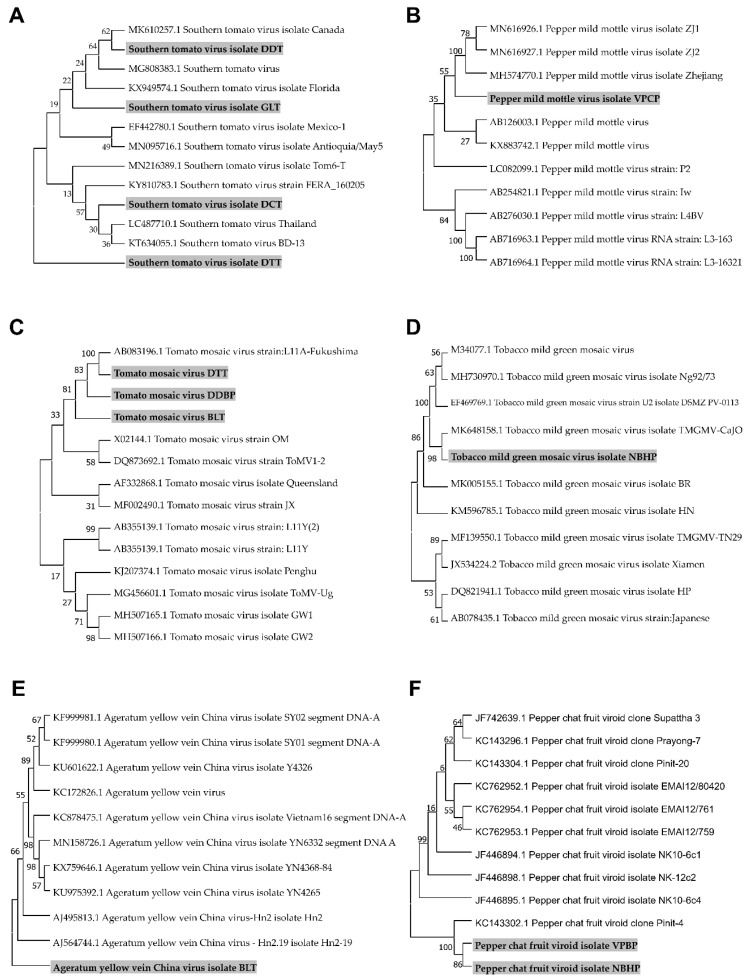
Phylogenetic trees for the identified five viruses and a viroid. For the construction of phylogenetic trees, we selected five viruses, including STV (**A**), PMMoV (**B**), ToMV (**C**), TMGMV (**D**), and AYVCNV (**E**), and a viroid PCFVd (**F**), in which genome sequences were assembled by RNA-Seq. Assembled viral genome sequences, as well as matched known viral genome sequences from GenBank, were subjected to the construction of phylogenetic trees. Phylogenetic trees were constructed using the MEGA7 program using the Maximum Likelihood method with a bootstrap of 1000 replicates. The identified viral isolates from this study were marked by the gray color.

We assembled viral genomes for four STV isolates, one PMMoV isolate, three ToMV isolates, one TMGMV isolate, one AYVCV isolate, and two PCFVd isolates by RNA-Seq and conducted bioinformatics analyses. The assembled viral genomes, which were assembled to complete genomes, were subjected to BLASTN search to retrieve homologous viral genome sequences. After nucleotide sequence alignment, we generated six different phylogenetic trees (Figure 6). The phylogenetic tree for STV isolates revealed that isolates including GLT (MW012410) and DDT (MW012413) were closely related, while STV isolates DCT (MW012412) and DTT (MW012411) were grouped in the other clade. Notably, STV isolate DTT was classified into different groups, in contrast to other isolates (Figure 6A). The identified PMMoV isolate VPCP (MW012414) was closely related to other isolates from China (Figure 6B). In the case of ToMV, we have already reported three partial genome sequences of ToMV from tomato, pepper leaves, and chili seeds [17]. The ToMV sequence from the previous study was used for phylogenetic tree construction. The phylogenetic tree of ToMV showed two distinct groups. Three ToMV isolates (isolate DTT; MW012409, isolate DDBP; MH393623, isolate BLT; MH393621) from Vietnam were grouped with an isolate from Japan (Figure 6C). The TMGMV isolate NBHP (MW012408) was closely related to the isolate CaJO from Jordan (Figure 6D). In the case of AYVCNV, we previously reported the assembled genome sequence as Ageratum yellow vein virus (AYVV) isolate BaoLoc [10]. However, a BLASTN search against a nucleotide database using the same identical genome sequence showed that the nearly complete genome for AYVV isolate BaoLoc showed strong genetic relationship with other known isolates from China (Figure 6E). Therefore, we renamed the virus as AYVCNV isolate BaoLoc (MW012407). Two PCFVd isolates (MW012406 and MW012415) from Vietnam were closely related with an isolate identified in tomato plants in Thailand (Figure 6F).

### 2.8. Validation of Results for RNA-Seq by RT-PCR

**Figure 7:**
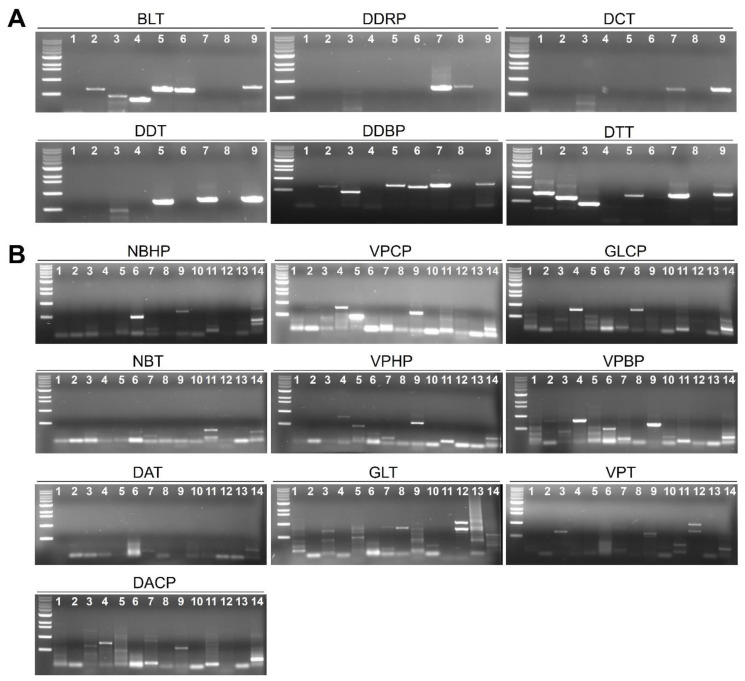
Confirmation of identified viruses and viroids by RT-PCR. RT-PCR results of viruses and viroids identified from the Southern (**A**) and Northern area (**B**) of Vietnam. Each sample on the gel was indicated by different number as follows (for panel A; 1-PVY, 2-CMV, 3-ToMV, 4-AVYCNV, 5-TYLCKaV, 6-CaCV, 7-TNRV, 8-PCV2, 9-STV, for panel B; 1-ChiVMV, 2-PVY, 3-CMV, 4-PCV2, 5-PMMoV, 6-TMGMV, 7-STV, 8-TNRV, 9-PeVYV, 10-ToLCVV, 11-TYLCVNV, 12-LAYVV, 13-CLVd, 14-PCFVd).

We carried out RT-PCR using virus-specific primers to confirm results of RNA-Seq. Virus-specific primers were designed based on the identified sequence for individual virus and viroid (Table 1). The results of RT-PCR were similar to those of RNA-Seq (Figure 7). For example, infection of two viruses—TNRV and PCV in the DDRP library—was confirmed by RT-PCR. In addition, we validated infection of six viruses in the DTT library using RT-PCR.

## 3. Discussion

Recently, a large number of viruses and viroids infecting tomato and pepper plants have been identified in a single study based on diverse NGS techniques [23,24,25]. Although tomato and pepper plants are widely cultivated in Vietnam and viruses infecting tomato and pepper plants can cause devastating epidemics, little is known about viruses and viroids infecting both plants. In this study, we identified 15 and 14 viral pathogens infecting tomato and pepper plants, respectively, grown in the diverse fields in Vietnam by RNA-Seq.

A previous study identified a total of 22 viruses infecting tomato derived from 170 field-grown samples in China by small RNA sequencing [25]. When we compared our results to the previous study, six viruses, including PVY, ChiVMV, CMV, ToMV, STV, and TYLCV, were commonly identified in both studies. In contrast, six viral pathogens, including AYVCNV, CaCV, PCSV, TNRV, CLVd, and LAYVV, were identified only in our study. Of these, with the exception of AYVCNV, this is the first report of five viral pathogens, including CaCV, PCSV, TNRV, CLVd, and LAYVV, infecting tomato plants in Vietnam. In addition, this is the first report of PCSV, TNRV, and LAYVV infecting tomato in the world.

Pepper viromes in two different pepper cultivars grown in India have previously been reported, revealing diverse DNA and RNA viruses infecting pepper plants [23]. Interestingly, none of the viruses were identified in both studies, suggesting geographical regions and plant varieties might be important factors for virus diversity. Both tomato and pepper plants are usually cultivated from seeds. In particular, seed transmission of several DNA viruses, such as TYLCV and pepper yellow leaf curl Indonesia virus, in pepper and chili pepper, respectively, have previously been reported [26,27]. Moreover, the seed transmission of viruses infecting tomato has been reported by several research groups [6,28,29]. In addition, seed transmission of two viroids, tomato planta macho viroid and PCFVd, in plants belonging to the family *Solanaceae* has been reported [8]. By contrast, a recent study demonstrated that TYLCV, known as a seed-transmitted begomovirus, was not seed transmitted in tomato and tobacco plants [30]. This study examined infection of TYLCV in surface-disinfected or untreated seeds, resulting in no infection of TYLCV, suggesting that most of the virus was located externally as a contaminant of the seed coat [30]. Regardless of seed-borne or seed transmission of several viruses and viroids infecting tomato and pepper plants, the seed could be a main factor for virus transmission in tomato and pepper plants in Vietnam.

The advance of NGS techniques facilitates the easy identification of known and unreported viruses in a target plant [21]. Based on RNA-seq, we were able to identify several unreported viruses and viroids in Vietnam. For example, this is the first study reporting eight viruses, including PVY, ChiVMV, CMV, CaCV, PCSV, PCV-2, TNRV, and TMGMV, as well as two viroids, CLVd and PCFVd, infecting pepper in Vietnam. Furthermore, we were able to determine the proportion of individual viral pathogen in a given sample and distribution of identified viruses and viroids in different regions and plants, as shown in the previous studies [31,32].

It is noteworthy that tomato and pepper plants are hosts for a wide range of viruses and viroids, as shown previously [23,25]. In our study, we identified a total of 18 viral species in eight genera. Moreover, the identified viruses and viroids have different kinds of genome types, such as ssDNA, dsRNA, ssRNA, and circular ssRNA. Of these, viruses with ssDNA genomes were frequently identified, suggesting that they could be major factors causing viral diseases in both plants, as suggested previously [33]. In the case of begomoviruses with ssDNA genomes, a different kind of begomovirus was identified in each region. Two viruses, PCV-2 and STV with dsRNA genome, were frequently identified in pepper and tomato plants, respectively. In fact, detailed disease symptoms caused by viruses with dsRNA genome have not been well characterized [34,35].

Furthermore, many viruses and viroids were commonly identified in both tomato and pepper plants, which are members in the family *Solanaceae* [36,37,38]. Based on our results, geographical region and host were important factors in determining viral population. For example, three libraries, DDT, DDBP, and DDRP, originated from different hosts but from the same region, Don Duong. The composition of viral pathogens in the three libraries was very similar, and PCSV was dominantly present in all three libraries. In the case of NBT and NBHP libraries, from tomato and pepper plants, respectively, grown in Ninh Binh, CLVd was dominant in NBT, while ChiVMV was dominant in NBHP, suggesting host-specific viral populations. We carefully supposed that the dominance of CLVd in NBT library might be associated with seed transmission of CLVd, as reported previously [9]. Some viruses, such as PVY from BLT, were associated with NGS reads, but not detected with PCR. RNA-Seq results demonstrate the number of virus-associated contig and coverage of sequence against the complete genome of each virus. To validate the RNA-Seq analysis using the conventional RT-PCR technique, a high level of sequence coverage is necessary. Although PVY in BLT library was identified by RNA-Seq with high values of viral-associated contigs, the coverage of PVY in BLT was low compared to other viruses. This disagreement between RNA-seq and RT-PCR suggests that we need to carefully check the level of coverage for successful validation by conventional RT-PCR, even though the number of virus-associated contigs was high.

Multiple infection by diverse viruses in a single host is very common. Similarly, at least seven different viral pathogens were found to infect both tomato (BLT) and pepper plants (DDBP). Furthermore, the analysis of viral population using virus-associated reads revealed that there was, preferentially, a dominant viral pathogen in tomato and pepper plants grown in the same field. However, we could not confirm whether the dominant viral pathogen played a major role in causing typical virus symptoms. Taken together, we identified diverse viruses and viroids infecting tomato and pepper plants grown in the fields in Vietnam by RNA-Seq. Our results showed that infection by different viral pathogens was common in the two plants. However, a specific viral pathogen was dominantly present, depending on the host plants as well as on the isolated regions, suggesting that the geographical region and host plant were two major factors for determining viral populations. Since we could not confirm whether the dominant virus identified by RNA-Seq is the major pathogen in each plant to cause viral symptoms observed, further experiments and screenings for identified viruses are required to provide more information for preventing virus diseases epidemic in pepper and tomato fields. Although we did not provide direct evidence for economic losses caused by virus infections in pepper and tomato plants in Vietnam, our results provide the comprehensive overview of viral pathogens infecting two important plants in the family *Solanaceae*. Many plant viruses reported in this study could infect diverse host plants and thus present the possibility of the continuous virus disease epidemics in the fields of Vietnam and potential threat to the agricultural industry.

## 4. Materials and Methods

### 4.1. Sample Collection and RNA Sequencing

Leaf samples were collected from plants exhibiting viral symptoms in open fields of pepper and tomato in Vietnam. We pooled leaf samples collected from the same geographical regions and host plants. Description of samples used in this study was were summarized in Table 2. Leaf samples were ground using pestle and mortar in the presence of liquid nitrogen. Total RNAs were extracted using RNeasy plant mini kit (Qiagen, Hilden, Germany). Extracted total RNAs were subjected to library preparation using NEBNext Ultra RNA Library Prep Kit for Illumina according to manufacturer’s instruction (NEB, Ipswich, MA, U.S.A.). Detailed library preparation is described in the previous study [39]. The prepared libraries were paired-end (2 × 100 bp) sequenced by HiSeq2000 system (Macrogen, Seoul, Korea).

### 4.2. Bioinformatic Analyses

Raw sequence files from each library were de novo assembled by the Trinity program with default parameters [40]. The assembled contigs from each library were subjected to a BLASTN search against viral genome database of National Center for Biotechnology Information (NCBI). The obtained virus-associated contigs were again subjected to a BLASTX search against NCBI’s non-redundant protein (NR) database to eliminate endogenous virus-like sequences. Finally, we identified viruses infecting tomato and pepper plants based on virus-associated contigs. We mapped raw sequence reads on the reference genomes of identified viruses using the Burrows–Wheeler Aligner (BWA) program with default parameters (http://bio-bwa.sourceforge.net/). The number of mapped reads for identified virus was calculated using bbmap.sh implemented in BBMap program (https://jgi.doe.gov/data-and-tools/bbtools/bb-tools-user-guide/bbmap-guide/). The raw data are available at the NCBI database with the BioProject number PRJNA636575.

### 4.3. Construction of Phylogenetic Trees

To generate phylogenetic trees, the assembled genome sequence of each virus was subjected to a BLASTN search against NCBI’s GenBank. We retrieved the top ten homologous viral genome sequences for individual virus or viroid. Nucleotide sequences were aligned by ClustalW program [41]. Phylogenetic trees were constructed by MEGA7 program using the maximum likelihood method based on the JTT matrix-based model with a bootstrap of 1000 replicates [42].

### 4.4. Reverse Transcription–Polymerase Chain Reaction (RT-PCR) to Validate Infection of Identified Viruses and Viroids

RT-PCR was carried out using virus-specific primers (Table 2). The RT-PCR reaction was conducted using the Diastar^TM^ Onestep RT-PCR kit (SolGent, Daejeon, Korea) following conditions based on the manufacturer’s instructions. The cycling conditions were 50 °C for 30 min, 95 °C for 15 min, followed by 30 cycles at 95 °C for 20 sec, 50 °C for 40 sec, and 72 °C for 1 min, with a final extension at 72 °C for 5 min. The PCR products were confirmed by gel electrophoresis with 1Kb DNA marker (Bioneer, Daejeon, Korea) in 1% agarose gel with TAE buffer.

## Figures and Tables

**Table 1:** Information of Primer Pairs Used for RT-PCR to Validate the RNA-Seq Results.

Primers *	Nucleotide Sequence	Expected Size
PVY_diag_F	ATGGCAAATGACACAATCGATGCAG	804 bp
PVY_diag_R	TCACATGTTCTTAACTCCAAGTAGAGTATG	
CMV_diag_F	ATGGACAAATCTGAATCAACCAGTG	754 bp
CMV_diag_R	GACTGGGAGCACTCCAGATG	
ToMV_diag_F	CGAGAGGGGCAACAAACAT	317 bp
ToMV_diag_R	ACCTGTCTCCATCTCTTTGG	
AYVCNV_diag_F	CTGATGTGCCCAAAGGTTGT	406 bp
AYVCNV_diag_R	GCCTGCTCCTTAGACGCATA	
TYLCKaV_diag_F	TGCCGAAGCGTTCAATAGAT	733 bp
TYLCKaV_diag_R	TGTTGCATACACAGGATTAGAGG	
CaCV_diag_F	AAGACCTCGAAAGAGGCAAA	703 bp
CaCV_diag_R	CTTCGGAGGCAAACTATTGG	
TNRV_diag_F	TTGCTAGCTGGAGGAGAAGC	786 bp
TNRV_diag_R	TCCTCTCCTAGTTGGCTTGC	
PCV2_diag_F	TTCAATCGACGGTTTCACAA	792 bp
PCV2_diag_R	CCTTGACTTGAGGTCGTGGT	
STV_diag_F	CAAAGGGAAGACTGCTGAGG	808 bp
STV_diag_R	AGCCTCTCCATCGGGATTAT	
ChiVMV_diag_F	GCGTAAAAGGCGAAGACTCA	782 bp
ChiVMV_diag_R	GTGCCGTTCAGTGTCCTCTT	
PMMoV_diag_F	ATGGCTTACACAGTTTCCAGTGCCAA	474 bp
PMMoV_diag_R	TTAAGGAGTTGTAGCCCAGGTGAGTCC	
TMGMV_diag_F	ATGCCTTATACAATCAACTCTCCG	480 bp
TMGMV_diag_R	CTAAGTAGCCGGAGTTGTGGTC	
PeVYV_diag_F	ATGAATACGGGAGGGGTTAGG	621 bp
PeVYV_diag_R	CTATTTCGGGTTGTGCAATTGC	
LaYVV_diag_F	ATGTCGAAGCGACCTGCAGATAT	741 bp
LaYVV_diag_R	GATTTTCAGAGTAGCATACACGGGA	
CLVd_diag_F	CGGAACTAAACTCGTGGTTCCTG	370 bp
CLVd_diag_R	AGGAACCTACTGCGGTTCCA	
PCFVd_diag_F	CCGGATTCTTCTAAGGGTGCCT	317 bp
PCFVd_diag_R	AGATCCTCTCGGGTCCCGG	
ToLCVV_diag_F	GCGTTAATGCGTCCCATAAT	528 bp
ToLCVV_diag_R	GCATTAAAGTCGTGGGCAAT	
TYLCVNV_diag_F	AGAAACGCCAAGTCTGAGGA	314 bp
TYLCVNV_diag_R	GTTCGGAGACGGAGAGTTGA	

* F and R indicate forward and reverse primer, respectively.

**Table 2:** Detailed Information for RNA-Seq Libraries and RNA-Seq Results.

Region *	Host Plant	Library	Total Read Bases (bp)	Total Reads	GC (%)
Dong Anh	Tomato	DAT	5,592,948,326	55,375,726	39.75
Gia Lam	Tomato	GLT	6,595,539,572	65,302,372	42.59
Vinh Phuc	Tomato	VPT	6,078,200,604	60,180,204	42.64
Ninh Binh	Tomato	NBT	4,899,434,654	48,509,254	41.2
Bao Loc city	Tomato	BLT	6,453,511,150	63,896,150	42.39
Duc Trong	Tomato	DTT	8,004,172,432	79,249,232	42.7
Don Duong	Tomato	DDT	7,918,928,432	78,405,232	43.8
Dalat	Tomato	DCT	8,257,251,768	81,754,968	43.67
DongAnh	Chili pepper	DACP	5,522,312,764	54,676,364	41.07
Gia Lam	Chili pepper	GLCP	4,912,867,452	48,642,252	41.24
Vinh Phuc	Chili pepper	VPCP	5,229,970,486	51,781,886	40.54
Vinh Phuc	Bell pepper	VPBP	5,745,072,910	56,881,910	42.45
Don Duong	Bell pepper	DDBP	8,473,848,288	83,899,488	44.83
Vinh Phuc	Hot pepper	VPHP	5,498,428,890	54,439,890	42.86
Ninh Binh	Hot pepper	NBHP	6,356,471,966	62,935,366	42.25
Don Duong	Red pepper	DDRP	8,339,452,436	82,568,836	43.24

* Geographical regions, host plants, and library names were described. Total read bases, total reads, GC percentage for each library by RNA-Seq were also provided.

## Supplementary Materials

The following are available online at https://www.mdpi.com/1422-0067/21/20/7565/s1. Table S1: Detailed information of identified viral pathogens (Virus and viroid) infecting tomato and pepper plants in different regions in Vietnam. Table S2: Number of virus-associated reads for identified viral pathogens.
